# Computed Tomography-Based Evaluation of Redo-Transcatheter Aortic Valve Replacement Feasibility for Self-Expanding Valves

**DOI:** 10.1016/j.shj.2025.100486

**Published:** 2025-05-29

**Authors:** Yusuke Kobari, Arif A. Khokhar, Davorka Lulic, Klaus Fuglsang Kofoed, Andreas Fuchs, Gintautas Bieliauskas, Sahil Khera, Gilbert HL. Tang, Ole De Backer

**Affiliations:** a Department of Cardiology, The Heart Center, Rigshospitalet, Copenhagen, Denmark; b Department of Clinical Medicine, University of Copenhagen, Copenhagen, Denmark; c Mount Sinai Fuster Heart Hospital, New York, New York, USA; d Department of Cardiovascular Surgery, Mount Sinai Health System, New York, New York, USA

**Keywords:** Aortic stenosis, Computed tomography, Lifetime management, Reintervention, Transcatheter aortic valve replacement

## Abstract

**Background:**

Redo-transcatheter aortic valve replacement (TAVR) may be unfeasible due to the risk of coronary flow compromise by the pinned-back leaflets of the index transcatheter aortic valve (TAV). This study aimed to evaluate the feasibility of redo-TAVR using a balloon-expandable SAPIEN 3 (S3, Edwards Lifesciences, USA) implanted within the intra-annular self-expanding Navitor TAV (Abbott, USA).

**Methods:**

A total of 106 post-TAVR computed tomography scans of patients who underwent Navitor implantation were analyzed. Redo-TAVR using an S3 was simulated in 2 positions: S3 outflow to node 2 of the Navitor (low implant) and S3 outflow to the base of the Navitor commissural posts (high implant). The overall coronary risk, determined by the risk of coronary flow compromise and coronary inaccessibility, was determined by the height of the neoskirt plane and the valve-to-aorta distances.

**Results:**

At a low S3 implant position, the overall coronary risk was high for only 1% of patients, but this increased to 39% with a high S3 implant position. If the high S3 implant was combined with a high index Navitor implant depth, 73% of patients were deemed high coronary risk, which could be reduced to 28% in case of an index Navitor implant depth >5 mm. At both S3 implant depths, redo-TAVR in Navitor was associated with a lower coronary risk compared to redo-TAVR in supra-annular self-expanding valves.

**Conclusions:**

The feasibility of redo-TAVR following S3-in-self-expanding valve depends on the type and implant depth of the index TAV as well as the implant depth of the second TAV.

## Highlights

• Redo-transcatheter aortic valve replacement (TAVR) feasibility depends on the type and implant depth of the index and second transcatheter aortic valve.
• Intra-annular vs. supra-annular index self-expanding valve has a more favorable profile for redo-TAVR.
• Leaflet modification techniques may impact redo-TAVR feasibility but require more study.

## Graphical abstract

Coronary risk classification for redo-TAVR in different self-expanding TAVs. Redo-TAVR was deemed unfeasible following a high S3 implant position for 39% of patients when the index TAV was a Navitor, 60% when the index TAV was an ACURATE neo2, and 75% when the index TAV was an Evolut. Lowering the S3 implant to a low position, predicted redo-TAVR unfeasibility for only 1% of patients with the Navitor, 8% with the ACURATE neo2, and 20% with the Evolut TAV. Abbreviations: S3, SAPIEN 3; TAV, transcatheter aortic valve; TAVR, transcatheter aortic valve replacement.

**Figure:**
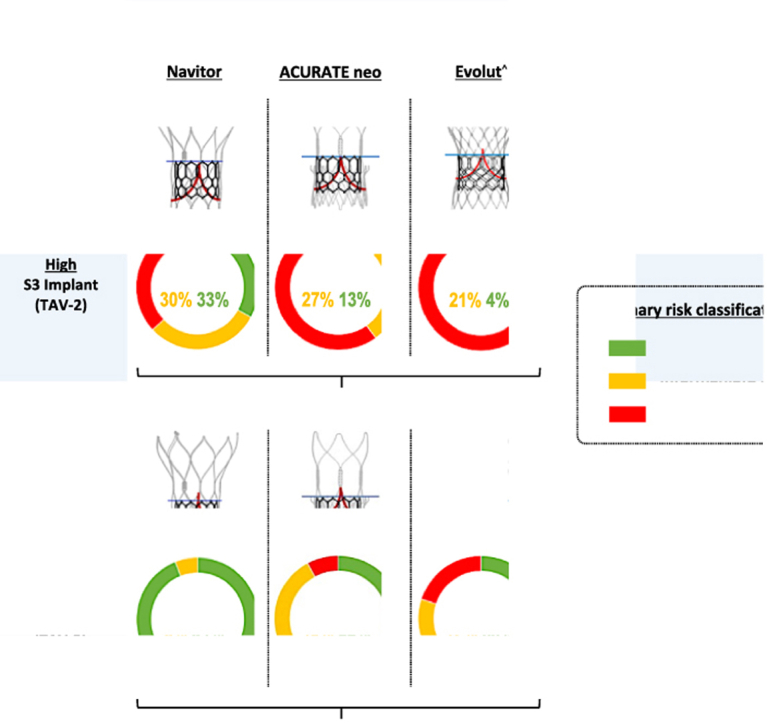

## Introduction

As transcatheter aortic valve replacement (TAVR) expands to younger populations, an increasing proportion of patients are expected to outlive the durability of their implanted transcatheter aortic valve (TAV).^1^^,^^2^ Although redo-TAVR compares favorably to surgical explantation, a significant proportion of redo-TAVR procedures are deemed unfeasible due to the risk of compromising coronary flow.^3^, ^4^, ^5^, ^6^, ^7^ This coronary risk is dictated by the pinned-up leaflets of the index TAV, termed neoskirt, and its relationship with the surrounding aortic root.^8^^,^^9^ Tall-frame supra-annular TAVs with their higher-positioned leaflets create a taller neoskirt, which increases the coronary risk during redo-TAVR.^10^^,^^11^

The Navitor (Abbott, USA) TAV is a tall-frame self-expanding valve (SEV) with its leaflets located in an intra-annular position.^12^ This lower position of the leaflets may confer an advantage when performing redo-TAVR in a Navitor using a balloon-expandable valve (BEV); however, clinical data remain limited.^13^^,^^14^

In this study, we used real-world post-TAVR computed tomography (CT) scans to evaluate the feasibility of redo-TAVR using the balloon-expandable SAPIEN 3 (S3, Edwards Lifesciences, USA) to treat a degenerated Navitor and compared the outcomes to prior real-world cohorts evaluating different combinations of S3-in-SEV.^15^

## Methods

### Study Population

Among 721 patients who underwent TAVR with the Navitor TAV across 2 centers, 106 patients had high-quality postimplant cardiac CT scans available, which were analyzed for this study. Patients treated with a Navitor TAV for a degenerated surgical or transcatheter bioprosthesis were excluded. All cardiac CT scans were electrocardiographically gated, contrast enhanced, and had a 0.5 mm slice thickness. CT analysis was performed using the 3Mensio imaging software (Pie Medical Imaging, the Netherlands), and all measurements were performed and verified independently by 2 experienced physicians (Y.K. and D.L.). Ethical approval for the study was granted by the local ethical committees, and written informed consent was waived given the study's retrospective design.

### Second TAV Positioning and Sizing

**Figure 1:**
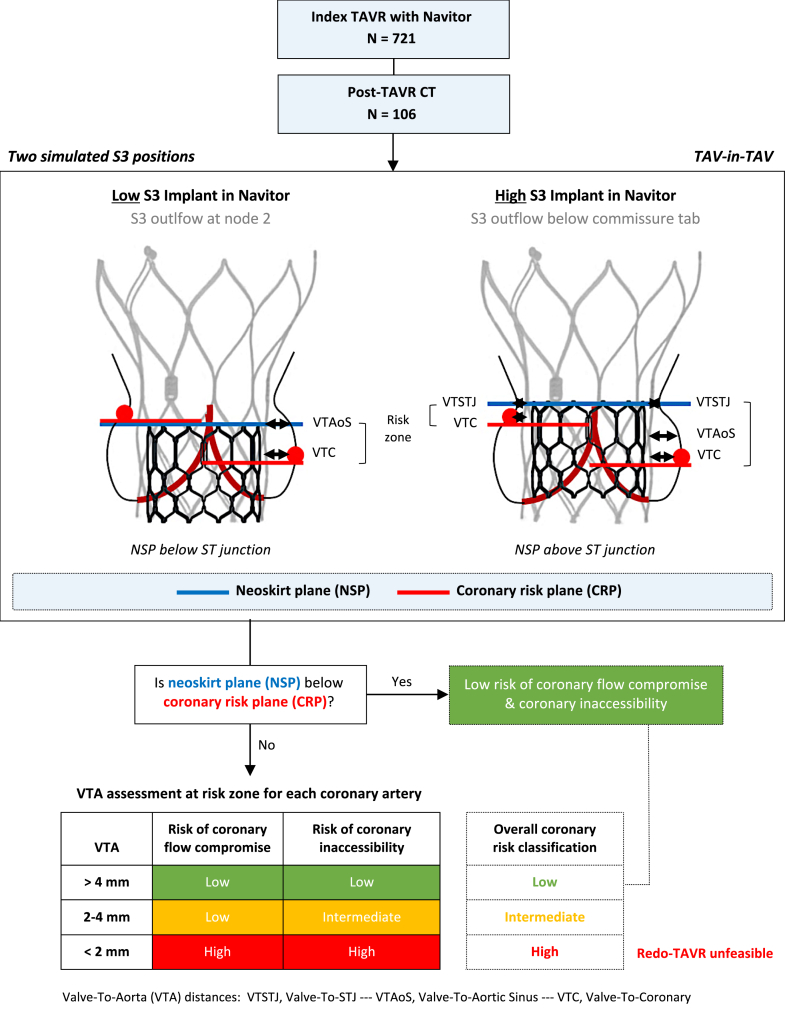
Study concept and methodology. The methodology used to evaluate the risk of coronary flow compromise and coronary inaccessibility, determining the overall coronary risk, following virtual S3 implantation in a Navitor index TAV using post-index TAVR CT scans. Definitions of the NSP and CRP can be found in the Methods section and the Redo TAV app. Abbreviations: CRP, coronary risk plane; CT, computed tomography; NSP, neoskirt plane; S3, SAPIEN 3; ST, sinotubular.; STJ, sinotubular junction; TAV, transcatheter aortic valve; TAVR, transcatheter aortic valve replacement; VTA, valve-to-aorta; VTAoS, valve-to-aortic sinus; VTC, valve-to-coronary; VTSTJ, valve-to-STJ

Post-TAVR CT scans were used to implant a virtual S3 inside the index Navitor TAV. Two different implant positions of the S3-in-Navitor were evaluated: for a low S3 implant, the outflow of the S3 was positioned in line with node 2 of the Navitor, and for a high S3 implant, the outflow was positioned just below the commissure tab of the Navitor (Figure 1).

For the main study analysis, sizing of the virtual S3 was based on the dimensions at the inflow portion of the index Navitor as measured on the post-TAVR CT scan (averaging the Navitor dimensions at inflow, node 1, node 2 for a low S3 implant and at node 1, node 2, and commissural level for a high S3 implant). This approach of in vivo sizing takes into consideration any potential frame underexpansion, which may impact the sizing strategy during redo-TAVR.^16^, ^17^, ^18^ An additional analysis was performed with S3 sizing based on the native annular dimensions obtained from the preindex TAVR CT scan; the results of this additional analysis are reported in the Supplementary File. In this way, the simulations cover the 2 extremes for S3 sizing. To mitigate for potential blooming artifact on CT, all dimensions were measured from the middle of the stent struts.

### Determining the Risk of Compromising Coronary Flow or Coronary Access

The risk of compromising coronary flow or coronary access was determined by evaluating the geometry between the simulated S3-in-Navitor redo-TAV complex and the surrounding aortic anatomy. Two planes were identified: the neoskirt plane (NSP) and the coronary risk plane (CRP).^19^ The NSP describes a plane parallel to and at the level of the S3 outflow and represents the height of the pinned-up leaflets of the index Navitor TAV during redo-TAVR. The higher the S3 implant, the higher the leaflets are pinned up, and the taller the NSP. The CRP was defined as the plane parallel to the aortic annulus at the lower margin of the coronary ostia and was obtained for both the left and right coronary ostia.

In a short-axis view, the linear distance between the simulated redo-TAV complex (S3-in-Navitor) and the surrounding aortic wall was measured in millimeters and termed valve-to-aorta (VTA) distance. Depending on the anatomy, the VTA can be evaluated at 3 different levels: (1) valve-to-coronary, (2) valve-to-aortic sinus, and (3) valve-to-sinotubular junction (STJ). VTA measurements were obtained for both the left and right coronary arteries (LCA/RCA). The subsequent relationship between the NSP, CRP, and VTA was then evaluated to determine the risk of compromising coronary flow (low/high) and coronary access (low/intermediate/high) for both coronary arteries.

Redo-TAVR for an S3-in-Navitor was deemed to be low risk for both coronary flow compromise and coronary inaccessibility if the NSP arose below the CRP.^19^ If the NSP arose above the CRP, then the narrowest of the 3 VTA measurements was used to further define the risk. The risk of coronary flow compromise was deemed to be low if the VTA was >2 mm and high risk if the VTA was <2 mm. For coronary access, the risk of inaccessibility was deemed to be low if the VTA was >4 mm, intermediate risk (or challenging) if the VTA was 2 to 4 mm, and high-risk for inaccessibility in case of VTA <2 mm.^19^ An overall coronary risk classification was then determined based on the risk of both coronary flow compromise and coronary inaccessibility. Coronary risk was classified as low risk when both coronary flow compromise and coronary inaccessibility were low risk, intermediate risk when coronary flow compromise was low risk but coronary inaccessibility was intermediate risk, and high risk when both coronary flow compromise and coronary inaccessibility were high risk (Figure 1). A coronary risk classification was obtained for both LCA and RCA, with the higher risk for either coronary determining the overall risk level for each patient.

### Statistical Analysis

Categorical variables are expressed as numbers (percentages), and continuous variables as medians (interquartile range). A two-sided chi-square test and Bonferroni correction to control the occurrence of false positives were used to compare the coronary risk classification for redo-TAVR in different self-expanding index TAVs—namely, the Navitor (Abbott, USA), ACURATE (Boston Scientific, USA), and Evolut (Medtronic, USA) SEVs as index TAVs. A two-sided *p*-value <0.05 was considered statistically significant. All statistical analyses were performed using SPSS version 29.0 (IBM, NY, USA).

## Results

For this study, 106 patients with a post-TAVR cardiac CT following Navitor implantation were included. Baseline clinical, CT, and procedural characteristics and in-hospital outcomes are summarized in Table 1. The median age of the study cohort was 79 (76-84) years, 44% were female, and the median Society of Thoracic Surgeons risk score was 4.4% (3.0%-6.7%). A size 23, 25, 27, 29, and 35 mm Navitor TAV was implanted in 6 (5.7%), 22 (20.8%), 28 (26.4%), 43 (40.6%), and 7 (6.6%) patients, respectively. Pre- and post-dilatation was performed in 84% and 49% of TAVR procedures, respectively.

**Table 1:** Baseline characteristics and in-hospital outcomes

	N = 106
Clinical characteristics	
Age, y	79 (76-84)
Sex, Female	47 (44.3%)
Arterial hypertension	75 (70.8%)
Diabetes mellitus	24 (22.6%)
Prior PCI	18 (17.0%)
Prior CABG	3 (2.8%)
Atrial fibrillation	25 (23.6%)
Prior stroke	9 (8.5%)
Peripheral arterial disease	12 (11.3%)
Reduced renal function∗	5 (4.7%)
STS risk score, %	4.4 (3.0-6.7)
Index CT anatomic characteristics	
Aortic annulus perimeter, mm	77.9 (71.2-81.9)
Aortic annulus area, mm^2^	462 (382-520)
Sinus of Valsalva, mean diameter, mm	31.8 (29.4-34.1)
STJ, mean diameter, mm	28.1 (25.3-30.0)
STJ, height, mm	22.4 (20.1-25.0)
Left coronary artery, height, mm	14.4 (12.0-16.2)
Right coronary artery, height, mm	17.6 (15.6-19.7)
TAVR procedure	
Navitor	
23-25 mm	28 (26.4%)
27 mm	28 (26.4%)
29 mm	43 (40.6%)
35 mm	7 (6.6%)
Predilatation	89 (84.0%)
Implant depth, mm	5.0 (3.7-6.3)
Postdilatation	52 (49.1%)
In-hospital outcomes	
Death	0 (0%)
Stroke	2 (1.9%)
Major vascular complication	2 (1.9%)
Permanent pacemaker implantation	12 (11.3%)
Predischarge echocardiographic outcomes	
Transprosthesis mean gradient, mmHg	8.0 (6.0-10.0)
Paravalvular regurgitation	
None-trace	56 (52.8%)
Mild	44 (41.5%)
Moderate/severe	5 (4.7%)

*Notes*. Data presented as n (%) or median (interquartile range).

Abbreviations: CABG, coronary bypass graft surgery; CT, computed tomography; IQR, interquartile range; PCI, percutaneous coronary intervention; STJ, sinotubular junction; STS, Society of Thoracic Surgeons; TAVR, transcatheter aortic valve replacement.

∗ Estimated glomerular filtration rate <30 mL/min/1.73 m^2^.

### Coronary Risk Based on S3 Implant Depth

**Figure 2:**
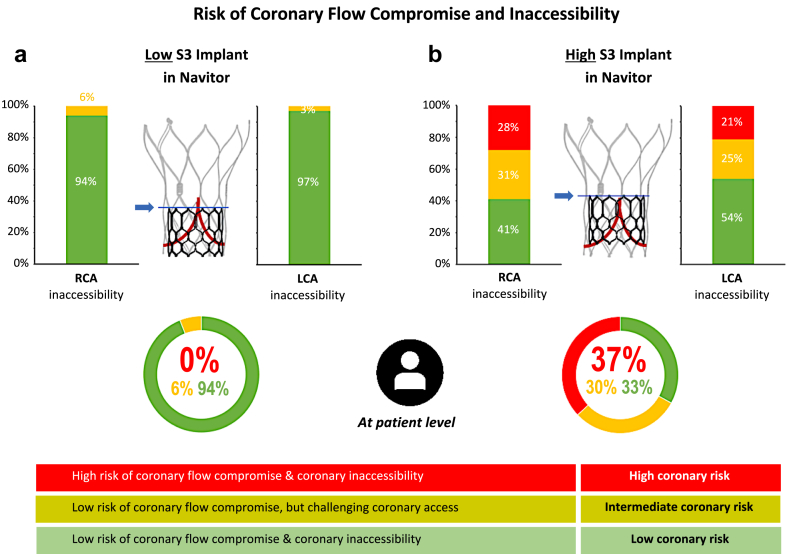
CT predicted risk of coronary flow compromise and coronary inaccessibility following redo-TAVR with S3-in-Navitor. (a) With a low S3 implant position targeting node 2 of the Navitor, only 1% of patients were deemed to be at high coronary risk, whereas (b) with a high S3 implant targeting the base of the commissural posts, 39% of patients were deemed to be at high coronary risk. Abbreviations: CT, computed tomography; LCA, left coronary artery; RCA, right coronary artery; S3, SAPIEN 3; TAVR, transcatheter aortic valve replacement.

The predicted risk of coronary flow compromise and coronary inaccessibility was dependent upon the implantation depth of the S3, with a greater risk observed with a higher S3 implant. At a high S3 implant, 37% of patients were deemed to be at high coronary risk (high risk for coronary flow compromise and coronary inaccessibility), 30% of patients at intermediate coronary risk (low risk for coronary flow compromise but with a challenging coronary access), and 33% of patients were predicted to be at low coronary risk (low risk for coronary flow compromise and coronary inaccessibility) (Figure 2).

Lowering the S3 implant depth resulted in 94% of patients being deemed at low coronary risk, while 6% and 0% of patients were deemed at intermediate and high coronary risk, respectively. Irrespective of the S3 implant depth, the risk for both coronary flow compromise and coronary inaccessibility was greatest for the RCA compared to the LCA. Similar results were observed when S3 sizing was based on the native annular dimensions obtained from the preindex TAVR CT scan (Supplementary Figure S1).

### Coronary Risk Based on Navitor Implant Depth

**Figure 3:**
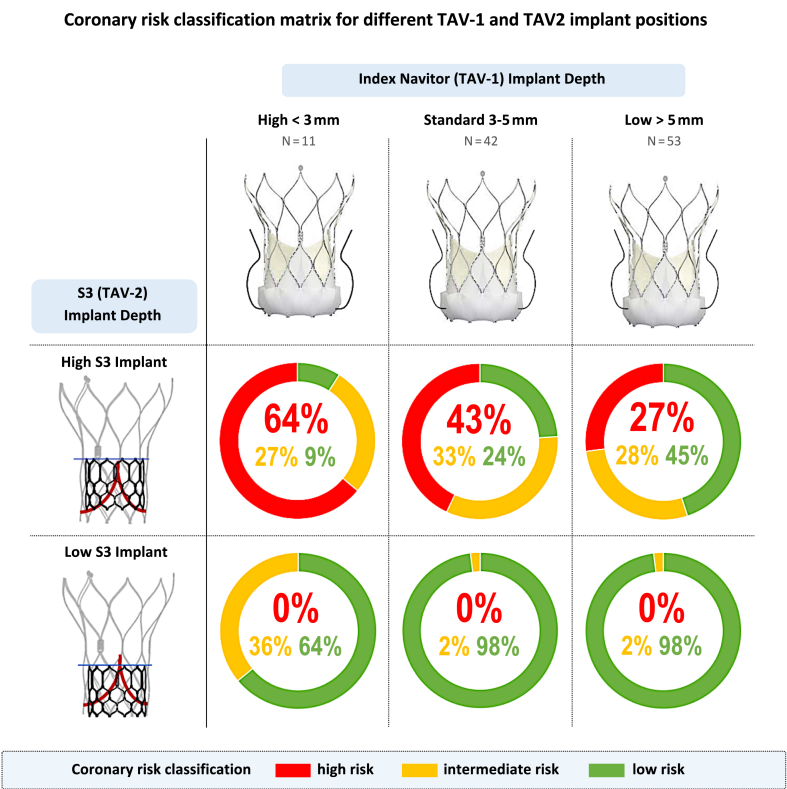
Coronary risk classification matrix for different TAV-1 and TAV-2 implant depths. The higher the implant depth of the index Navitor TAV, the greater the coronary risk in case of redo-TAVR. A high index Navitor implant combined with a high S3 position predicted a high coronary risk for 73% of patients, which was reduced to 2% with a low Navitor implant and low S3 position. Abbreviations: S3, SAPIEN 3; TAV, transcatheter aortic valve; TAVR, transcatheter aortic valve replacement.

A standard implant depth (3-5 mm) of the Navitor was achieved in 39.6% of patients (N = 42), with a high implant (<3 mm) in 10.3% (N = 11) and a low implant (>5 mm) in 50% (N = 53) of patients. As shown in Figure 3, the higher the implant depth of the index Navitor TAV, the greater the risk of compromising coronary flow or coronary access in case of redo-TAVR. In the worst-case scenario of a high index Navitor implant (<3 mm) combined with a high S3 implant, the coronary risk was deemed to be high in 64% of patients. In the best-case scenario of a standard or low index Navitor implant (>3 mm) combined with a low S3 implant, 98% of patients were deemed to be at low coronary risk. Similar results were observed when S3 sizing was based on the native annular dimensions obtained from the preindex TAVR CT scan (Supplementary Figure S2).

## Discussion

In this study, we used post-TAVR CT scans to evaluate and compare the real-world feasibility of redo-TAVR for a degenerated Navitor using an S3 at 2 different implant depths. The key conclusions are (1) redo-TAVR with S3-in-Navitor was deemed feasible for 94% of patients if the S3 was implanted low, (2) altering the implant depth of the S3 has a significant impact on the coronary risk when planning for redo-TAVR, (3) the implant depth of the index Navitor TAV significantly affects potential feasibility for redo-TAVR, and (4) S3-in-Navitor was associated with the lowest coronary risk in comparison to other S3-in-SEV combinations.

### Implant Depth of Second TAV

When implanting a short-frame BEV inside a Navitor, the leaflets of the index TAV are pinned back, creating a covered barrier termed neoskirt.^8^^,^^10^ The height of this neoskirt and its relationship to the coronary ostia and STJ dictate the coronary risk after redo-TAVR.^11^^,^^19^ In our study, 37% of patients were deemed high-risk for coronary flow compromise or inaccessibility following a high implantation of the S3. In contrast, lowering the S3 implantation to align the outflow at node 2 of the Navitor resulted in 0% of patients being deemed high risk for coronary flow compromise or inaccessibility (Figure 2). These results are aligned with prior CT-based simulation studies evaluating redo-TAVR feasibility using an S3 inside tall-frame self-expanding TAVs.^5^, ^6^, ^7^ A real-world analysis of post-TAVR CT scans with the ACURATE neo2 demonstrated that with a high S3 implant, 60% of patients would be at high risk for coronary flow compromise and coronary inaccessibility, and this risk could be lowered to just 8% with a low S3 implant.^19^ Similarly, analysis of post-TAVR CT scans derived from the Low Risk Evolut trial demonstrated that 75% of patients had the highest risk of compromising coronary flow and access with a high S3 implant, which reduced down to 20% of patients deemed low risk with a low S3 implant.^6^^,^^7^ These data demonstrate the potential benefit of using a BEV to treat tall-frame TAVs, whereby operators can modulate the neoskirt height by adjusting the implant depth of the BEV, directly impacting the coronary risk and potential feasibility of the redo-TAVR procedure. For further practical considerations on how to plan and perform redo-TAVR across different clinical scenarios, we recommend referring to the Redo TAV app in conjunction with recent state-of-the-art publications on this topic.^19^^,^^20^

### Impact of Index TAV on Redo-TAVR Feasibility

Prior CT-based studies have shown that the choice of index TAV can influence the subsequent feasibility of redo-TAVR, with a BEV-first strategy associated with potentially more favorable coronary access, reduced need for leaflet modification, and increased feasibility for redo-TAVR.^5^^,^^21^ By comparing the outcomes of 3 studies, which used the same methodology, we are able to compare the coronary risk following redo-TAVR with different combinations of BEV-in-SEV and this based on real-world post-TAVR CT scan analyses.^6^^,^^15^ The risk of compromising coronary flow and coronary inaccessibility with a high S3 implant has been reported to be 37%, 60%, and 75% for S3-in-Navitor, S3-in-ACURATE neo2, and S3-in-Evolut, respectively. A low S3 implant rendered 0% of patients high risk in case of S3-in-Navitor, whereas this remained 8% and 20% with S3-in-ACURATE neo2 and S3-in-Evolut, respectively (*p* < 0.001; Graphic Abstract). Irrespective of the S3 implant depth, the S3-in-Navitor combination is associated with the lowest coronary risk due to its intra-annular leaflet position, which results in a shorter neoskirt height. In contrast, for supra-annular SEVs, particularly when the S3 is implanted in a high position, the resulting neoskirt height is more likely to extend beyond the coronary ostia and/or STJ, risking compromising coronary flow or coronary access.

In addition to TAV selection, altering the target implant depth of the index TAV can also impact the future risk and feasibility of redo-TAVR. In our study, the higher the implant depth of the index Navitor, the greater the subsequent coronary risk after redo-TAVR. If an implant depth of <3 mm was achieved, then redo-TAVR with a high S3 implant predicted a high coronary risk for 64% of patients. If the index Navitor was implanted at the target 3 to 5 mm or even lower at >5 mm, then a high coronary risk was predicted for 43% or 27% of patients, respectively (Figure 3). Similar findings were reported from the Evolut Low-Risk CT substudy, which compared the feasibility of coronary access following S3-in-Evolut at 2 different implant depths of the index Evolut. If the index Evolut was implanted at 3 mm, then a high S3 implant would render coronary access unfeasible to both ostia in 61% of patients. If the index Evolut was implanted at 5 mm, then unfeasible coronary access was predicted for 31% of patients.^7^

Taken together, these results highlight how an operator can influence the coronary risk and potential feasibility for redo-TAVR at the time of index TAVR. When using a tall-frame SEV as the first valve, the unique design elements of the Navitor TAV with its intra-annular leaflets and larger open cells appear favorable when considering the coronary risks following redo-TAVR. Additionally, the relatively straighter inflow portion of the valve allows an operator to target a specific implant depth more precisely than with other SEV platforms. With an Evolut prosthesis, achieving a deeper implantation can risk the valve diving toward the left ventricular outflow tract due to the tapered design of the waist, which becomes more prominent with the larger valve sizes. For an ACURATE neo2, the final implant depth achieved is often dictated by the length of the calcified leaflets and therefore cannot be precisely controlled by an operator.

### Study Limitations

There are several limitations to note in this study. Firstly, the patients did not undergo an actual redo-TAVR procedure; therefore, the CT-based predictions may not fully reflect the physiological conditions in real-world procedures. Also, the simulations may not fully capture the *in vivo* expansion of the Navitor or S3 valves that may occur during redo-TAVR; as a consequence, this may also have an impact on the estimated coronary risk in this study. Secondly, not all patients who underwent TAVR with the Navitor valve could be included in this study, but only those with interpretable high-quality post-TAVR CT scans, potentially introducing selection bias. Also, the comparison of outcomes—in this case, coronary risk classification—across different studies may carry the risk of introducing selection bias; however, it is reassuring to note that the index CT anatomic characteristics are comparable across the 3 compared studies (Graphic Abstract). Finally, additional adjunctive procedures and techniques that may influence coronary flow and coronary access after redo-TAVR, such as commissural alignment or leaflet modification, were not considered due to limited data and experience.

## Conclusions

When planning for TAVR in a young low-risk patient, the feasibility and options for redo-TAVR should always be considered. This study indicates that redo-TAVR feasibility following S3-in-SEV depends on the type and implant depth of the index TAV as well as the implant depth of the second TAV. The choice for an index SEV with intra-annular leaflet position has a more favorable profile for redo-TAVR as compared to SEVs with supra-annular leaflet position.

## Ethics Statement

Ethical approval for the study was granted by the local ethical committees.

## Funding

The authors have no funding to report.

## Disclosure Statement

Y. Kobari has received financial support from the Japan Heart Foundation for his fellowship and speaker fees from Abbott Structural Heart and Boston Scientific. A.A. Khokhar received speaker fees from Boston Scientific. G. Bieliauskas received institutional research grants and consulting fees from Boston Scientific. S. Khera is a consultant and proctor for Medtronic, a consultant and proctor for Abbott Structural Heart, a consultant and proctor for W. L. Gore & Associates, a consultant for Terumo, a consultant and advisory board member of EastEnd Medical, and serves on the speaker’s bureau for Zoll Medical and Edwards Lifesciences. G.H.L. Tang has received speaker's honoraria and served as a physician proctor, consultant, advisory board member, TAVR publications committee member, RESTORE study steering committee member, APOLLO trial screening committee member, and IMPACT MR steering committee member for Medtronic, has received speaker's honoraria and served as a physician proctor, consultant, advisory board member, and TRILUMINATE trial anatomic eligibility and publications committee member for Abbott Structural Heart, has served as an advisory board member for Boston Scientific and JenaValve, a consultant and physician screening committee member for Shockwave Medical, a consultant for NeoChord, Peija Medical, and Shenqi Medical Technology, and has received speaker's honoraria from Siemens Healthineers. O. De Backer received institutional research grants and consulting fees from Abbott, Boston Scientific, and Medtronic. The other authors had no conflicts to declare.
