# Supplementary material for: Computed Tomography-Based Evaluation of Redo-Transcatheter Aortic Valve Replacement Feasibility for Self-Expanding Valves
Source: Struct Heart. 2025 May 29;9(10):100486. doi: 10.1016/j.shj.2025.100486 (PMC12455090; doi:10.1016/j.shj.2025.100486)
Supplement: Supplementary file [file mmc1.pdf]

Figure S1

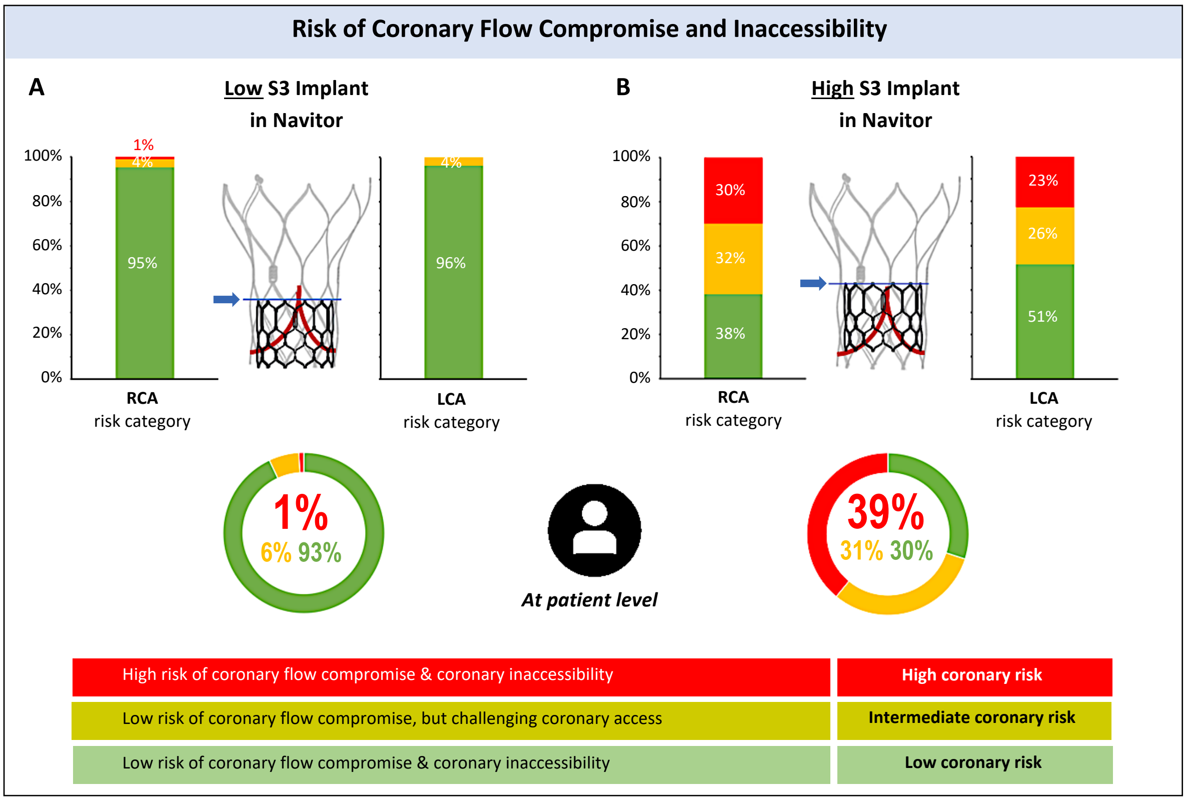

Figure S1. CT predicted risk of coronary flow compromise and coronary inaccessibility following redo-TAVR with Sapien 3 (S3)-in-Navitor, with S3 sizing based on Navitor inflow dimensions. When S3 sizing was determined based on the inflow portion of the Navitor on post-TAVR CT, **(A)** no patients were deemed to have a high coronary risk with a low S3 implant position targeting node 2 of the Navitor, whereas **(B)** 37% of patients were deemed to be at high coronary risk with a high S3 implant targeting the base of the commissural posts. CT, computed tomography; TAVR, transcatheter aortic valve replacement.

Figure S2

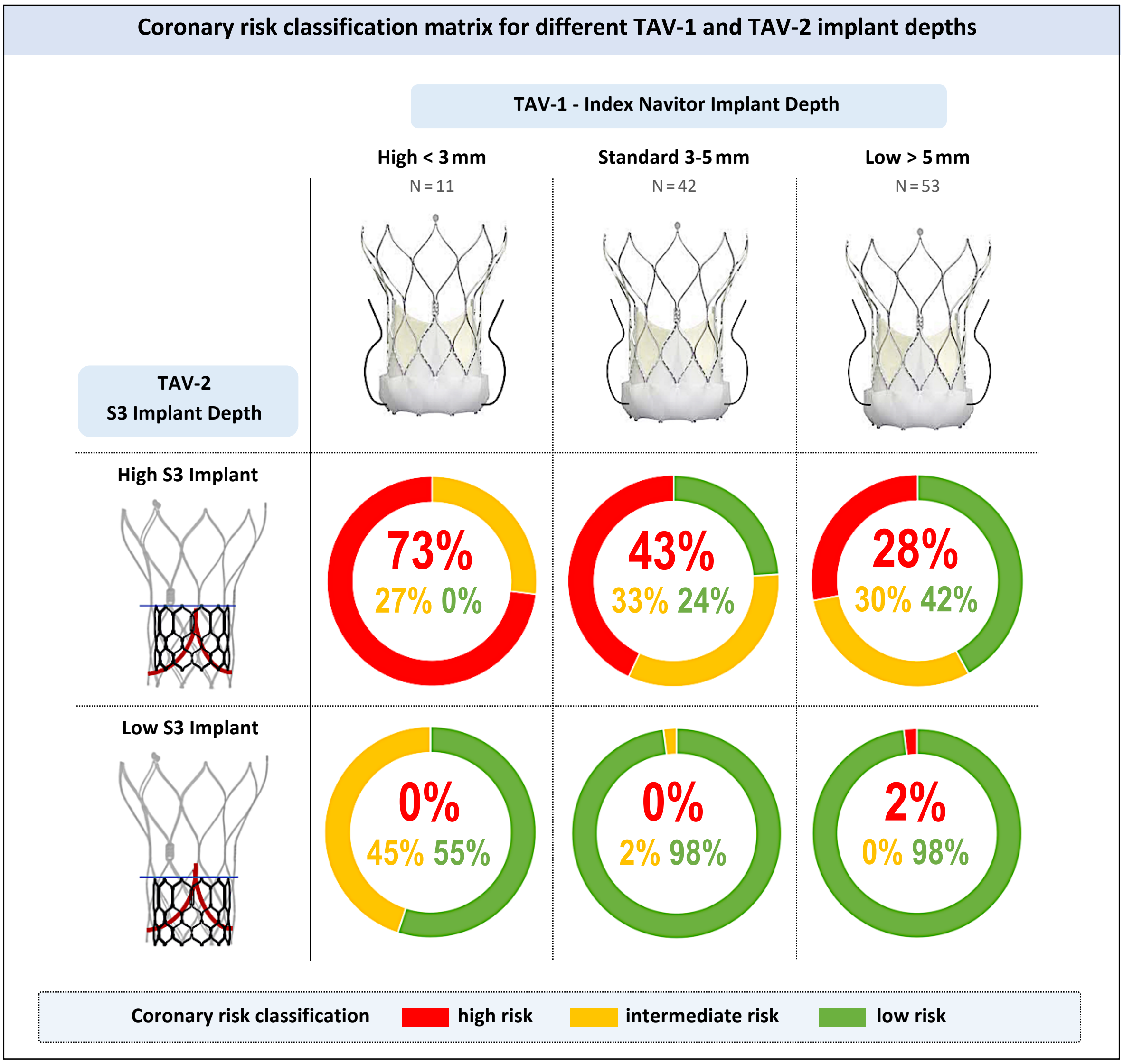

Figure S2. Coronary risk classification matrix for different TAV-1 and TAV-2 implantation depths. The higher the implant depth of the index Navitor TAV, the greater the coronary risk in case of redo-TAVR. A high index Navitor implant combined with a high S3 position for redo-TAVR predicted a high coronary risk for 73% of patients, which was reduced to 2% with a low Navitor implant and low S3 position. TAV, transcatheter aortic valve; TAVR, transcatheter aortic valve replacement.
